# Prognostic nutritional index as a predictor of in-hospital mortality in neonates and infants undergoing cardiac surgery

**DOI:** 10.3389/fnut.2025.1686989

**Published:** 2025-11-13

**Authors:** Xiaolin Gu, Jie Li, Xiaoqin Huang, Rongxing Bao, Hailin He, Liuyuan Li, Dandong Luo, Chongjian Zhang

**Affiliations:** Department of Cardiac Surgical Intensive Care Unit, Guangdong Provincial People's Hospital (Guangdong Academy of Medical Sciences), Southern Medical University, Guangzhou, China

**Keywords:** prognostic nutritional index, neonates, infants, congenital heart disease, cardiac surgery, mortality

## Abstract

**Objective:**

The prognostic nutritional index (PNI), combining nutritional and systemic inflammation markers is suggested as a predictor of negative outcomes post-cardiac surgery. This study investigated the link between PNI and in-hospital mortality in neonates and infants undergoing cardiac surgery.

**Methods:**

This retrospective cohort study included 3,082 neonates and infants (aged ≤ 365 days) who underwent primary cardiac surgery between January 2017 and October 2023. The PNI was utilized to evaluate nutritional status. Patients were stratified into two groups based on PNI values: low (PNI ≤ 51.65) and high (PNI > 51.65).The association between PNI and in-hospital mortality was assessed using multivariable logistic regression models, adjusted for demographic, preoperative, and surgical variables. Subgroup analyses were performed to assess potential effect modification. The potential linear relationship between PNI and mortality was examined using generalized additive models and smooth curve fitting.

**Results:**

The overall in-hospital mortality rate was 1.72% (53/3, 082). Patients with a PNI ≤ 51.65 exhibited a significantly higher mortality rate of 6.03% compared to 0.92% for those with a PNI > 51.65 (*p* < 0.001). Multivariable analysis identified PNI as an independent predictor of in-hospital mortality (adjusted odds ratio: 0.95; 95% CI: 0.91–0.98; *p* = 0.005). Subgroup analyses revealed significant effect modification by age, congenital heart disease (CHD) complexity, and cardiopulmonary bypass status (*p* for interaction < 0.05). The inverse relationship between PNI and mortality was stronger in neonates aged 30 days or younger, patients with non-complex CHD, and those undergoing off-pump surgery. Receiver operating characteristic analysis indicated that PNI effectively predicted in-hospital mortality, with an area under the curve of 0.745 (95% CI: 0.675–0.815; *p* < 0.001) and an optimal cutoff value of 51.65 determined by the Youden index.

**Conclusions:**

PNI independently predicts in-hospital mortality in neonates and infants undergoing cardiac surgery. The findings indicate that PNI could be an efficient tool for preoperative risk assessment in high-risk populations.

## 1 Introduction

Congenital heart disease (CHD) includes various structural cardiac anomalies formed during embryonic development (1). It is a major cause of infant mortality, surpassing all other congenital anomalies combined (2). This vulnerability is exemplified by the early onset of heart failure, a common and devastating complication that contributes substantially to the poor outcomes in infants with CHD (3). The immature myocardium in neonates and infants is particularly susceptible to pressure or volume overload, precipitating rapid clinical deterioration (4). Epidemiological data show an increasing incidence of CHD, now estimated at 8.98%, significantly threatening neonatal and infant health and survival. Surgical intervention is the primary definitive treatment. Despite advances in surgical techniques and perioperative care, neonates and infants undergoing cardiac surgery continue to face elevated risks of morbidity and mortality (5).

Malnutrition is a prevalent and critical comorbidity in this population, contributing significantly to adverse outcomes. Preoperative malnutrition is linked to higher perioperative complications, extended hospital stays, and diminished postoperative quality of life (6). It negatively impacts various organ systems, such as cardiovascular, immune, endocrine, and gastrointestinal, and hinders wound healing and recovery (7). Neonates and infants are especially susceptible due to limited nutritional reserves, lower muscle and fat mass, and high resting energy expenditure. In the setting of critical illness, this population is at heightened risk for protein-energy malnutrition (8).

The Prognostic Nutritional Index (PNI) is a validated tool extensively utilized to evaluate nutritional risk in adult surgical and oncologic populations. Initially created to assess perioperative risk in gastrointestinal surgery patients, the Prognostic Nutritional Index (PNI) has been utilized across diverse clinical settings (9). In oncology, reduced PNI scores are regularly linked to unfavorable outcomes (10). Recently, PNI has shown prognostic value in cardiovascular diseases, including heart failure, pulmonary embolism, and acute coronary syndrome, particularly in patients receiving percutaneous coronary intervention (11, 12). However, its application in pediatric populations is still underexplored. Previous research has explored PNI as a predictor of kidney function decline in children with chronic kidney disease and its link to mortality in pediatric cardiac surgery patients (*n* = 98) (13, 14). Due to limited evidence, this study examined the relationship between the PNI and in-hospital mortality among neonates and infants undergoing cardiac surgery. The results could enhance risk stratification and guide interventions to improve perioperative outcomes in this vulnerable group.

## 2 Materials and methods

### 2.1 Study design and population

**Figure 1:**
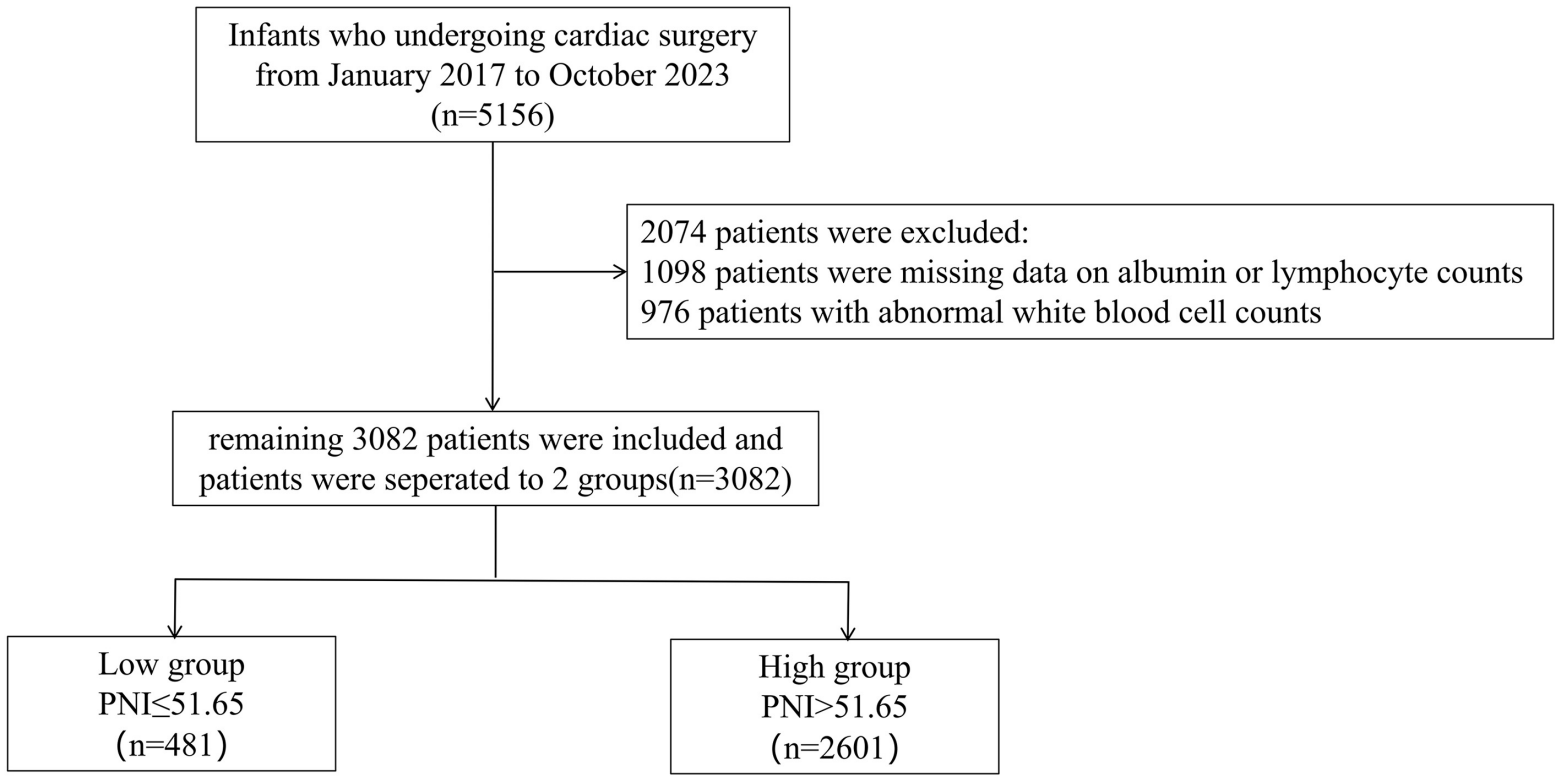
Outlines the patient enrollment process.

This retrospective cohort study analyzed neonates and infants (age ≤ 365 days) who underwent first-time cardiac surgery at Guangdong Provincial People's Hospital (Guangzhou, China) between January 2017 and October 2023. The initial screening included 5, 156 eligible patients. Of these, 2, 074 were excluded due to missing preoperative albumin or lymphocyte count data or abnormal white blood cell (WBC) count values (Figure 1). The final study cohort comprised 3, 082 patients. Patients were categorized into two groups according to their Prognostic Nutritional Index (PNI) values, with a cutoff of 51.65 optimized via the Youden index from ROC curve analysis (15). The low PNI group comprised patients with PNI ≤ 51.65 (*n* = 481), while the high PNI group included those with PNI > 51.65 (*n* = 2, 601). The primary outcome was in-hospital mortality. The Ethics Committee of Guangdong Provincial People's Hospital approved the study protocol (approval No. KY2024-605-02).

### 2.2 Data collection

Initial demographic and clinical information was obtained from the hospital's electronic medical records. Data collected encompassed age, sex, height, weight, the Society of Thoracic Surgeons-European Association for Cardio-Thoracic Surgery (STS-EACTS) score, comorbidities (cyanotic and complex CHD), and preoperative laboratory parameters such as serum albumin, total lymphocyte count, glucose, creatinine, WBC count, and hemoglobin. Complex CHD was defined as any congenital heart defect other than simple lesions as classified by the 2020 ESC/EACTS Guidelines, which include isolated small atrial or ventricular septal defects, patent ductus arteriosus, and mild pulmonary stenosis (16). Intraoperative variables such as cardiopulmonary bypass (CPB) duration and aortic cross-clamp time were documented. The Prognostic Nutritional Index (PNI) was determined using the formula: PNI = serum albumin (g/L) + [5 × total lymphocyte count (per mm3)] (17).

### 2.3 Statistical analysis

Continuous variables are presented as mean ± standard deviation. Categorical variables were presented as frequency (percentage). Continuous variables were compared using independent *t*-tests, while categorical variables were assessed with chi-square tests. For covariates with missing values in under 25% of samples, we utilized the Random Forest algorithm for imputation, effectively managing complex interactions and non-linear relationships. This was implemented via the Miss Forest approach, an iterative method enhancing imputation accuracy by treating missing data as a regression issue (18). To evaluate the relationship between PNI score and in-hospital mortality, both univariate and multivariate logistic regression models were developed, acknowledging that these variables might be part of the causal pathway between patients and in-hospital mortality. Consequently, we assessed the risk across various models. Model 1 is unadjusted. Model 2 includes adjustments for gender, age, gestational age, height, and weight. Model 3 incorporates additional adjustments for cyanotic CHD, complex CHD, STS-EACTS score, CPB time, aortic cross-clamp time, CPB status, hemoglobin, glucose, creatinine, and WBC. The potential linear association between PNI and in-hospital mortality was assessed using generalized additive models and smooth curve fitting. We performed a receiver operating characteristic (ROC) analysis to assess the predictive value of PNI for in-hospital mortality in patients. Subgroup analyses were performed to identify potential effect modifiers in the association between PNI and in-hospital mortality. Logistic regression was used to estimate associations, with results presented as odds ratios and 95% confidence intervals. Statistical analyses were conducted using R software (v4.1; R Foundation for Statistical Computing, http://www.R-project.org) and Empower Stats (v4.1; X&Y Solutions, Inc.). A two-sided *p*-value of < 0.05 was deemed statistically significant.

## 3 Results

### 3.1 Baseline patient characteristics

The study initially assessed 5,156 eligible participants for inclusion. Following thorough screening and evaluation, 3,082 participants were included in the study. Reasons for exclusion included missing data on albumin or lymphocyte count (*n* = 1,098), abnormal WBC count (*n* = 976). Abnormal WBC counts are defined as values below 4 × 10^9^/L or above 12 × 10^9^/L. Figure 1 outlines the patient enrollment process.

Table 1 presents the demographic characteristics of the participants. The study included 1,965 males and 1,117 females, with an average age of 146.79±92.09 days. Among the participants, 852 had cyanotic congenital heart disease (CHD), and 2976 had complex CHD. In-hospital mortality was 1.72% (53/3, 082). Patients were categorized into two groups based on PNI ( ≤ 51.65 and >51.65). Patients in the low PNI group had a significantly higher risk of in-hospital mortality than those in the high PNI group (6.03% vs. 0.92%, *p* < 0.01). Significant differences were observed between the groups in terms of age, height, weight, cyanotic CHD, albumin, lymphocyte count, glucose, creatinine, WBC, CPB, CPB time, aortic cross-clamp time, and STS-EACTS score. No significant differences were observed between the two groups with respect to sex, complex congenital heart disease, or hemoglobin levels.

**Table 1:** Baseline characteristics of patients stratified by the PNI.

**Characteristic**		**PNI**		
	**Total**	**Low**	**High**	***P*** **-value**
		≤ **51.65**	>**51.65**	
*N*	3,082	481	2,601	
Age (days)	146.79 ± 92.09	86.54 ± 78.32	157.94 ± 90.12	< 0.001
Gestational Age (weeks)	38.54 ± 2.10	37.83 ± 2.72	38.67 ± 1.93	< 0.001
**Gender**, ***n*** **(%)**				
Female	1,117 (36.24%)	160 (33.26%)	957 (36.79%)	0.139
Male	1,965 (63.76%)	321 (66.74%)	1,644 (63.21%)	
Height (cm)	60.16 ± 8.42	53.80 ± 7.16	61.34 ± 8.10	< 0.001
Weight (kg)	5.47 ± 1.76	4.10 ± 1.44	5.73 ± 1.69	< 0.001
**Cyanotic CHD**, ***n*** **(%)**				
No	2,230 (72.36%)	305 (63.41%)	1,925 (74.01%)	< 0.001
Yes	852 (27.64%)	176 (36.59%)	676 (25.99%)	
**Complex CHD**, ***n*** **(%)**				
No	106 (3.44%)	13 (2.70%)	93 (3.58%)	0.335
Yes	2,976 (96.56%)	468 (97.30%)	2,508 (96.42%)	
Albumin (g/L)	38.88 ± 4.30	33.24 ± 3.91	39.93 ± 3.48	< 0.001
Hemoglobin (g/L)	112.77 ± 18.00	111.75 ± 18.65	112.96 ± 17.88	0.173
Lymphocyte count (10^9^/L)	4.96 ± 1.75	2.60 ± 0.82	5.40 ± 1.51	< 0.001
Glucose (mmol/L)	5.16 ± 1.26	5.27 ± 1.76	5.14 ± 1.14	0.038
Creatinine (μmol/L)	26.15 ± 12.61	32.80 ± 20.51	24.92 ± 10.05	< 0.001
WBC (10^9^/L)	8.72 ± 1.83	7.69 ± 1.94	8.91 ± 1.75	< 0.001
PNI	63.70 ± 11.25	46.23 ± 4.62	66.93 ± 8.90	< 0.001
**CPB**, ***n*** **(%)**				
No	294 (9.54%)	70 (14.55%)	224 (8.61%)	< 0.001
Yes	2,788 (90.46%)	411 (85.45%)	2,377 (91.39%)	
CPB time (min)	86.69 ± 58.31	102.54 ± 78.44	83.76 ± 53.27	< 0.001
Aortic cross-clamp time (min)	46.49 ± 34.23	52.13 ± 44.82	45.44 ± 31.79	< 0.001
STS-EACTS score	2.60 ± 0.86	2.77 ± 0.94	2.57 ± 0.85	< 0.001
**In-hospital mortality**, ***n*** **(%)**				
No	3,029 (98.28%)	452 (93.97%)	2,577 (99.08%)	< 0.001
Yes	53 (1.72%)	29 (6.03%)	24 (0.92%)	

PNI, prognostic nutritional index; CHD, congenital heart disease; WBC, white blood cells; CPB, cardiopulmonary bypass; STS-EACTS score, Society of Thoracic Surgeons–European Association for Cardio-Thoracic Surgery.

### 3.2 Univariate logistic regression analysis

Table 2 presents univariate regression results, revealing PNI's significant association with in-hospital mortality. Factors associated with in-hospital mortality include age (OR 0.99, 95% CI 0.98–0.99, *p* < 0.0001), gestational age (OR 0.80, 95% CI 0.74–0.87, *p* < 0.0001), height (OR 0.89, 95% CI 0.86–0.93, *p* < 0.0001), weight (OR 0.56, 95% CI 0.46–0.69, *p* < 0.0001), cyanotic CHD (OR 3.5, 95% CI 2.02–6.06, *p* < 0.0001), albumin (OR 0.81, 95% CI 0.76–0.85, *p* < 0.0001), hemoglobin (OR 1.03, 95% CI 1.02–1.04, *p* < 0.0001), lymphocyte count (OR 0.63, 95% CI 0.53–0.75, *p* < 0.0001), glucose (OR 0.70, 95% CI 0.53–0.93, *p* = 0.012), creatinine (OR 1.04, 95% CI 1.02–1.05, *p* < 0.0001), PNI (OR 0.91, 95% CI 0.89–0.94, *p* < 0.0001), CPB time (OR 1.01, 95% CI 1.01–1.02, *p* < 0.0001), aortic cross-clamp time (OR 1.02, 95% CI 1.01–1.02, *p* < 0.0001), and STS-EACTS score (OR 2.34, 95% CI 1.61–3.40, *p* < 0.0001).

**Table 2:** Univariate Logistic regression analysis for in-hospital mortality of PNI.

**Variables**	**OR**	**(95%CI)**	***p*-value**
Age (days)	0.99	(0.98, 0.99)	< 0.0001
Gestational age (weeks)	0.80	(0.74, 0.87)	< 0.0001
**Gender**, ***n*** **(%)**			
Female	Ref		
Male	1.76	(0.94, 3.31)	0.077
Height (cm)	0.89	(0.86, 0.93)	< 0.0001
Weight (kg)	0.56	(0.46, 0.69)	< 0.0001
**Cyanotic CHD**, ***n*** **(%)**			
No	Ref		
Yes	3.50	(2.02, 6.06)	< 0.0001
**Complex CHD**, ***n*** **(%)**			
No	Ref		
Yes	0.43	(0.15, 1.21)	0.108
Albumin (g/L)	0.81	(0.76, 0.85)	< 0.0001
Hemoglobin (g/L)	1.03	(1.02, 1.04)	< 0.0001
Lymphocyte count (10^9^/L)	0.63	(0.53, 0.75)	< 0.0001
Glucose (mmol/L)	0.70	(0.53, 0.93)	0.012
Creatinine (μmol/L)	1.04	(1.02, 1.05)	< 0.0001
WBC (10^9^/L)	0.89	(0.77, 1.03)	0.112
PNI	0.91	(0.89, 0.94)	< 0.0001
**CPB**, ***n*** **(%)**			
No	Ref		
Yes	1.30	(0.46, 3.62)	0.619
CPB time (min)	1.01	(1.01, 1.02)	< 0.0001
Aortic cross-clamp time (min)	1.02	(1.01, 1.02)	< 0.0001
STS-EACTS score	2.34	(1.61, 3.40)	< 0.0001

PNI, prognostic nutritional index; CHD, congenital heart disease; WBC, white blood cells; CPB, cardiopulmonary bypass;STS-EACTS score, Society of Thoracic Surgeons-European Association for Cardio-Thoracic Surgery.

### 3.3 Multivariate logistic regression models

PNI demonstrated a significant association with in-hospital mortality when used as a continuous variable (change per 1 SD, OR: 0.95, 95% CI: 0.91–0.98, *p* = 0.005). When analyzed as a categorical variable, the high PNI group exhibited a 57% lower risk of in-hospital mortality compared with the low PNI group in model 3 (Table 3).

**Table 3:** Presents a multivariate logistic regression analysis examining the association between PNI and in-hospital mortality.

**Variables**	**Model 1**		**Model 2**		**Model 3**	
	**OR (95% CI)**	***p*** **-value**	**OR (95% CI)**	***p*** **-value**	**OR (95% CI)**	***p*** **-value**
PNI (Per 1SD)	0.91 (0.89, 0.94)	< 0.0001	0.94 (0.91, 0.96)	< 0.0001	0.95 (0.91, 0.98)	0.005
**PNI**						
≤ 51.65	Ref		Ref		Ref	
>51.65	0.15 (0.08, 0.25)	< 0.0001	0.28 (0.15, 0.53)	< 0.0001	0.43 (0.21, 0.88)	0.022

Model 1: no covariates were adjusted for.

Model 2: adjust for gender, age, gestational age, height, weight.

Model 3: adjust for Model 2 and cyanotic CHD, complex CHD, STS-EACTS score, CPB time, aortic cross-clamp time, CPB, preoperative hemoglobin, preoperative glucose, preoperative creatinine, preoperative WBC. PNI, prognostic nutritional index; CHD, congenital heart disease; WBC, white blood cells; CPB, cardiopulmonary bypass.

### 3.4 Subgroup analysis

Subgroup analyses were performed to identify potential effect modifiers in the association between PNI and the risk of in-hospital mortality. Stratification factors included gender, age, gestational age, cyanotic CHD, complex CHD, CPB and STS-EACTS score. The results indicated no significant differences across subgroups for gender, gestational age, cyanotic CHD, and STS-EACTS score (*p*>0.05). The upper limit of the OR was < 1.00 across all subgroups, suggesting an association between higher baseline patient characteristic PNI and decreased in-hospital mortality. The subgroup analysis revealed significant effect modification by age, CHD complexity, and CPB status (*p* for interaction < 0.05). Higher PNI was more protective against in-hospital mortality in neonates and infants ≤ 30 days old (OR 0.86, 95% CI 0.81–0.92), patients with non-complex CHD (OR 0.86, 95% CI 0.76–0.97), and those not undergoing CPB (OR 0.82, 95% CI 0.72–0.94) (Table 4).

**Table 4:** Effect size of PNI on In-hospital mortality in each subgroup.

**In-hospital Mortality**	***N***	**OR (95%CI)**	***p*-value**	***p* for interaction**
**Age (days)**				
≤ 30	275	0.86 (0.81, 0.92)	< 0.0001	0.001
>30	2,807	0.94 (0.91, 0.97)	< 0.0001	
**Gestational age (weeks)**				
≤ 37	643	0.89 (0.84, 0.93)	< 0.0001	0.174
>37	2,439	0.93 (0.90, 0.96)	< 0.0001	
**Gender**, ***n*** **(%)**				
Female	1,117	0.96 (0.91, 1.01)	0.0862	0.204
Male	1,965	0.90 (0.87, 0.93)	< 0.0001	
**Cyanotic CHD**, ***n*** **(%)**				
No	2,230	0.91 (0.88, 0.95)	< 0.0001	0.626
Yes	852	0.92 (0.88, 0.95)	< 0.0001	
**Complex CHD**, ***n*** **(%)**				
No	106	0.86 (0.76, 0.97)	0.0153	0.001
Yes	2,976	0.91 (0.89, 0.94)	< 0.0001	
**CPB**, ***n*** **(%)**				
No	294	0.82 (0.72, 0.94)	0.0038	0.034
Yes	2,788	0.92 (0.89, 0.94)	< 0.0001	
**STS-EACTS score**				
≤ 2	1,183	0.91 (0.86, 0.95)	< 0.0001	0.092
>2	1,899	0.92 (0.89, 0.94)	< 0.0001	

CHD stands for congenital heart disease, CPB refers to cardiopulmonary bypass;STS-EACTS score denotes the Society of Thoracic Surgeons - European Association for Cardio-Thoracic Surgery.

### 3.5 Receiver operating characteristic analysis

**Figure 2:**
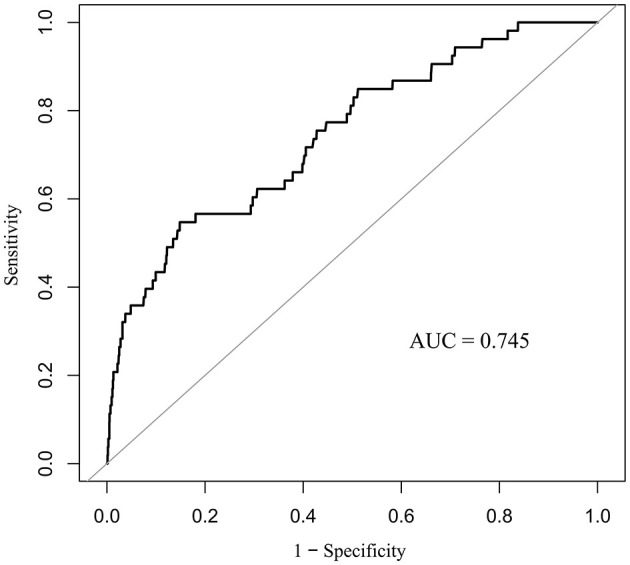
Receiver operating characteristic curves of the PNI and in-hospital mortality.

We constructed ROC curves (Figure 2) to assess the predictive value of PNI for in-hospital mortality in neonates and infants undergoing congenital heart disease surgery. The study found that the PNI had an area under the curve (AUC) of 0.745 (95% CI: 0.675–0.815, *p* < 0.001), with an optimal cutoff point of 51.65 based on the Youden index.

### 3.6 Association between PNI and in-hospital mortality

**Figure 3:**
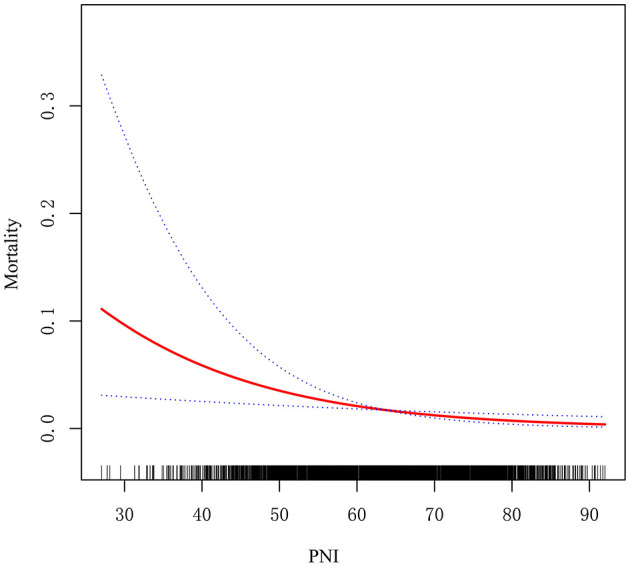
Association between the PNI and In-hospital Mortality.

The potential linear association between PNI and in-hospital mortality was assessed using generalized additive models and smooth curve fitting. The findings revealed a negative correlation between the PNI and in-hospital mortality, suggesting that higher PNI levels are associated with lower in-hospital mortality rates (Figure 3).

## 4 Discussion

This extensive retrospective cohort study revealed a notable inverse relationship between the Prognostic Nutritional Index (PNI) and in-hospital mortality among neonates and infants undergoing cardiac surgery for congenital heart disease (CHD). Patients with a PNI ≤ 51.65 had significantly higher mortality rates than those with a PNI >51.65. PNI was independently linked to in-hospital mortality after accounting for potential confounders. This study, the largest of its kind with 3, 082 participants, evaluates the prognostic utility of PNI in a high-risk pediatric population, filling a crucial gap in the literature. ROC analysis confirmed PNI's predictive value, showing an AUC of 0.745 and an optimal cutoff of 51.65 via the Youden index. Subgroup analyses indicated significant effect modification by age, CHD complexity, and CPB status. The link between PNI and mortality was particularly significant in neonates aged ≤ 30 days, patients with non-complex CHD, and those undergoing off-pump procedures. The findings indicate that PNI could be an effective biomarker for risk stratification in this vulnerable group.

According to the European Society for Clinical Nutrition and Metabolism, malnutrition arises from inadequate nutritional intake, affecting body composition and cellular mass, and impairing physical and mental functions. In surgical patients, preoperative protein-energy malnutrition is linked to higher postoperative morbidity and mortality, highlighting the importance of early nutritional assessment and intervention to improve perioperative outcomes (19).

The PNI integrates serum albumin (reflecting hepatic synthetic capacity and protein-energy balance) and total lymphocyte count (indicating cell-mediated immunity) into a composite biomarker. This synthesis quantifies three interdependent postoperative determinants: nutritional reserves, systemic inflammation, and immunocompetence. Both parameters derive from standard perioperative laboratory panels, eliminating need for specialized assays. Such operational pragmatism facilitates longitudinal tracking of nutritional-inflammatory dynamics across the surgical continuum—from preoperative risk stratification to postoperative convalescence assessment (20).

It is important to note that the PNI is fundamentally a marker intended to reflect chronic nutritional and immunologic reserves. In this study, we excluded patients with preoperative leukocytosis (WBC > 12 × 10?/L) or leukopenia (WBC < 4 × 10?/L) to mitigate the confounding effects of acute systemic inflammation. In neonates and infants, such abnormal WBC counts are highly indicative of active infection, significant surgical stress, or other inflammatory states. These acute-phase responses can simultaneously depress serum albumin synthesis and cause aberrant shifts in lymphocyte counts through mechanisms such as demargination or the release of immature granulocytes (21). Consequently, an acute inflammatory state can artificially inflate the PNI, creating a false impression of adequate nutrition in a patient who is actually under significant physiologic stress. By focusing our analysis on patients without overt preoperative inflammation, we aimed to ensure that the PNI values more accurately reflected baseline nutritional status rather than acute-phase distortions, thereby preserving the validity of our risk stratification (22).

However, despite having a well-proven prognostic value in adult cardiac surgery, PNI has yet to be studied comprehensively for its effect in infant cardiac surgery. Existing evidence on PNI's prognostic value in infant cardiac surgery remains fragmented and constrained by critical limitations. While prior studies predominantly linked low PNI to secondary outcomes such as prolonged ICU stay (Kaur et al., *n* = 108) or acute kidney injury (*n* = 108), only one small-scale study (*n* = 98) explicitly examined mortality, reporting no significant association (1, 14, 23). In contrast, our analysis of 3, 082 neonates and infants—the largest cohort to date—definitively establishes PNI as an independent predictor of in-hospital mortality. Crucially, we further identify neonates ≤ 30 days, non-complex CHD patients, and off-pump cases as high-sensitivity subgroups where PNI's predictive power is markedly enhanced. This granular risk stratification, previously unattainable in smaller studies, provides clinically actionable insights for targeted nutritional interventions.

It is important to recognize certain limitations. Initially, Because this study was conducted at a single center, the generalizability of our findings to other institutions may be limited. Second, our analysis was restricted to neonates and infants undergoing their first cardiac surgery, which may not be representative of older pediatric or adult populations. Third, although we identified a significant association between PNI and mortality, our study design precludes causal inference. The impact of perioperative nutritional support on clinical outcomes in patients with low PNI is yet to be established. Prospective multicenter studies and randomized controlled trials are necessary to confirm our findings and investigate the benefits of preoperative nutritional optimization in this population. Fourth, the exclusion of patients with abnormal white blood cell counts, a proxy for acute systemic inflammation, means that our derived PNI cutoff value and its associated risk estimates are most applicable to a preoperative population in a relatively stable, non-inflammatory state. The prognostic performance of the PNI in neonates and infants with concurrent active infection or significant inflammatory stress remains unclear and warrants further investigation, potentially through the development of an inflammation-corrected PNI model.

## 5 Conclusion

This extensive retrospective cohort study found an independent inverse relationship between PNI and in-hospital mortality among neonates and infants undergoing cardiac surgery. These findings underscore PNI's potential as a straightforward, accessible, and dependable biomarker for risk stratification in high-risk populations.

## Data Availability

The raw data supporting the conclusions of this article will be made available by the authors, without undue reservation.
